# Supplementary material for: Transport properties and doping evolution of the Fermi surface in cuprates
Source: Sci Rep. 2023 Aug 21;13:13562. doi: 10.1038/s41598-023-39813-z (PMC10442347; doi:10.1038/s41598-023-39813-z)
Supplement: Supplementary file 1 — Supplementary Information. [file 41598_2023_39813_MOESM1_ESM.pdf]

# Supplementary Information for: Transport properties and doping evolution of the Fermi surface in cuprates

**B. Klebel-Knobloch[1], W. Tabiś[2,1], M. A. Gala[1,2], O. S. Barišić[3,*], D. K. Sunko[4,*], and N. Barišić[1,4,*]**

[1]Institute of Solid State Physics, TU Wien, 1040 Vienna, Austria
[2]AGH University of Krakow, Faculty of Physics and Applied Computer Science, 30-059 Krakow, Poland
[3]Institute of Physics, Bijenička cesta 46, HR-10000, Zagreb, Croatia
[4]Department of Physics, Faculty of Science, University of Zagreb, Bijenička cesta 32, HR-10000, Zagreb, Croatia
[*]obarisic@ifs.hr, dks@phy.hr, nbarisic@phy.hr

## 1 LSCO – additional observations

In the main text, our discussion is focused on the doping levels $p < 0.30$. The reason is threefold. First, our main interest is to understand the strong deviation from the universal properties, which starts at $p \sim 0.08$ in LSCO. Second, we were interested in the evolution of $n_H$ in the $p$ to $1+p$ regime which occurs below $p < 0.27$. Third, the reported ARPES tight-binding parametrization extends to $p = 0.30$. Therefore, we decided to constrain our discussion conservatively.

However, measurements performed on thin films[1] show that $n_H$ becomes negative both at high doping ($p > 0.32$) and, even more instructively, rather abruptly upon increasing the temperature at $p = 0.32$, see Fig. S1. Both sign-reversals are easily captured within the proposed arc elongation with temperature/doping.

At high doping levels ($p > 0.35$), the Fermi surface (FS) is closed and electron-like, thus it is to be expected that $n_H$ carries a negative sign. To understand the abrupt change as a function of temperature, it is sufficient to note that the FS close to $p = 0.30$ still consists of arcs, with long, flat "metallic" segments that are dominantly hole-like, while the short electron-like segments at the end of the Brillouin zone are partially gapped.

Simply by looking at such a FS, it is easy to deduce that even a small arc elongation with temperature, encompassing the strongly curved electron-like segments, causes large changes in $n_H$, eventually provoking the observed sign-reversal. As shown in Fig. S1b, the results of our calculations reveal that $n_H$ indeed changes its sign to become negative at elevated doping levels. We also note that the calculated $n_H$ agrees with the reported values from Ref. 1 only quantitatively—which we attribute to difficulties of both synthesizing homogeneous LSCO films at high Sr concentrations and ascertaining the exact hole-content of the CuO layers.

We also find it instructive to calculate the $n_H$ of the (ungapped) underlying Fermi-surface Fig. S2. In Fig. S2, the difference between the $n_H$'s calculated from the pseudogapped (dashed line) and ungapped Fermi surface (full line) is a simple vertical shift by one elementary charge, straightforwardly related to the (de)localization of exactly one charge per $CuO_2$ plaquette. We find that the simplicity of this argument makes it hard to contest the reality of delocalization. Sometimes, a picture is worth a thousand words.

## 2 Universality of nodal $v_F$ and the absolute value of $\sigma_{xx}$

In this work, we use published best-fit parametrizations of ARPES data, in the form of tight-binding dispersions. Typically, the fit for such models takes into account energy windows which capture a significant portion of the band which crosses the Fermi level, or more generally the shape of the FS, not only the (low) energy scale which is relevant for transport processes. Therefore, the nodal Fermi velocities ($v_F^n$) at $< 30$ meV from the Fermi level are not always captured with high accuracy. Because the simple FL transport calculations are sensitive to the value of $v_F^n$, only tight-binding parametrizations that capture its observed value with sufficient precision can be used in direct comparisons with the experiment.

A careful analysis shows the $v_F^n$ (at $4\,\text{meV} < \omega < 30\,\text{meV}$) to be universal across a number of cuprate families and the relevant doping range (see Fig. S3a).[2–4] Because $v_F^n$ from the tight-binding fit to ARPES data of Hg1201 is closest to the observed universal value of $\sim$1.8 eV Å, in the calculation for $\tau\rho_\square$, we normalize the other compounds to Hg1201. The corresponding factors of normalization $f_{\text{norm}}$ are given in Fig. S3b. The corrected $\tau\rho_\square$ is shown in the main text (Fig. 4a), while the uncorrected one is in Fig. S3c. This normalization may be incorporated into the line integrals given by Eqs. (3) and (4) in the main text, applying a simple substitution $\varepsilon_{\vec{k}} \to f_{\text{norm}}\varepsilon_{\vec{k}}$. It is easy to see that such a rescaling of the dispersion keeps the values of the Hall coefficient $R_H$ unchanged, as given by Eq. (2) and shown in the figures in the main text.

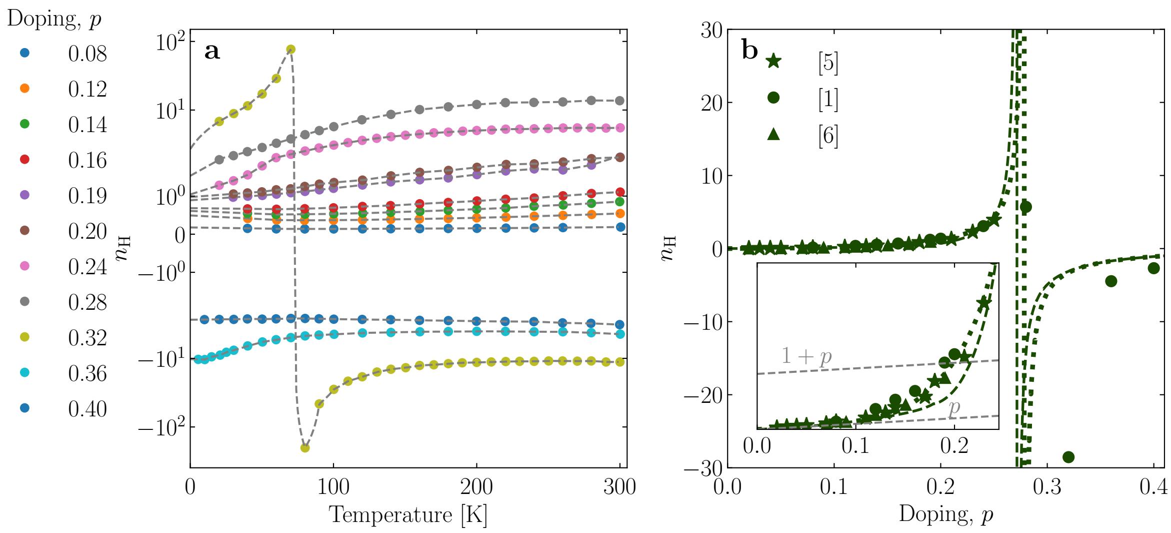


**Figure S1.** Temperature and doping dependence of $n_H$ in thin LSCO films. **a** Temperature dependence of $n_H$ for selected doping levels, shown on a logarithmic scale.[1] The points are measured data while grey dashed lines correspond to spline interpolations. Clearly, $n_H$ changes sign at $p \sim 0.32$. Moreover, at $p = 0.32$ the sign change appears as a function of temperature, between 70 K and 80 K. Such a dependence clearly reveals that the main temperature effect is a re-population of the arcs. The main consequence of the proximity of the Lifshitz transition is that, due to the flatness of the nodal segments, it emphasizes the re-population effect in $n_H$. **b** The Hall number of LSCO, as in Fig. 3c of the main text, in an extended doping range, showing the reentrance of $n_H$, both measured[1,5,6] and calculated, from the negative side. The inset shows a closeup of the low-doping range, discussed in the main text.

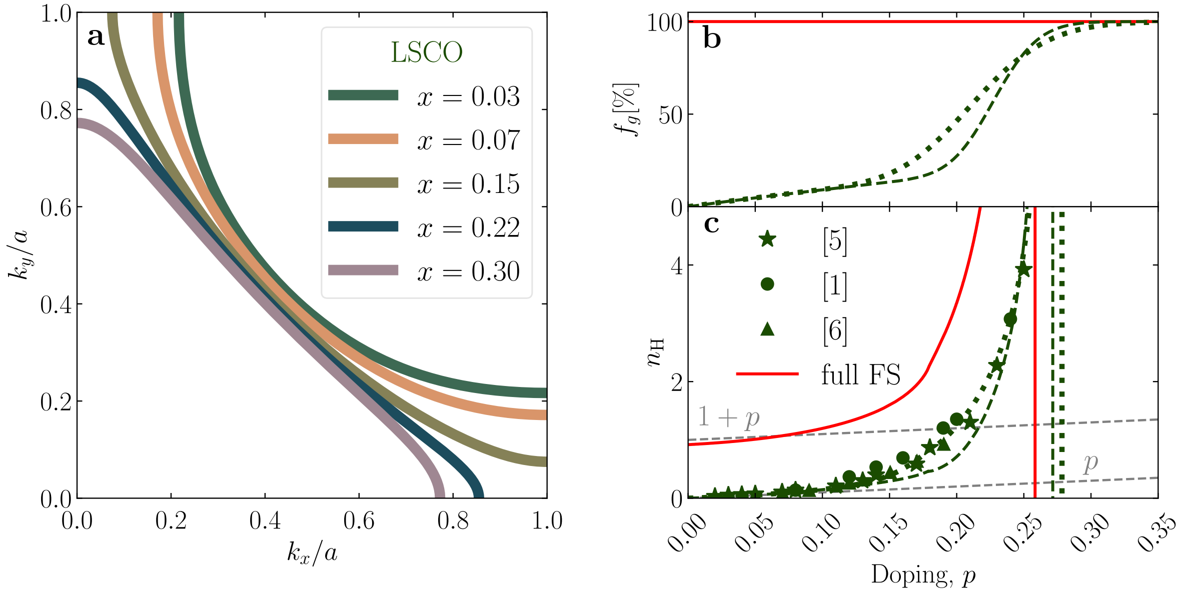


**Figure S2.** The underlying (ungapped) FS and Hall number in LSCO. In **a**, the underlying FS as parametrized in Ref. 7 is shown. In contrast to the pseudogapped case (see Fig. 3 in the main text), the fraction of ungapped states $f_g$ is now 100%, depicted in **b** as red line. **c** The calculated $n_H$ of the underlying FS is depicted with a red line here too, and compared to the real (pseudogapped) case, discussed in the main text and also shown in Fig. 3c.

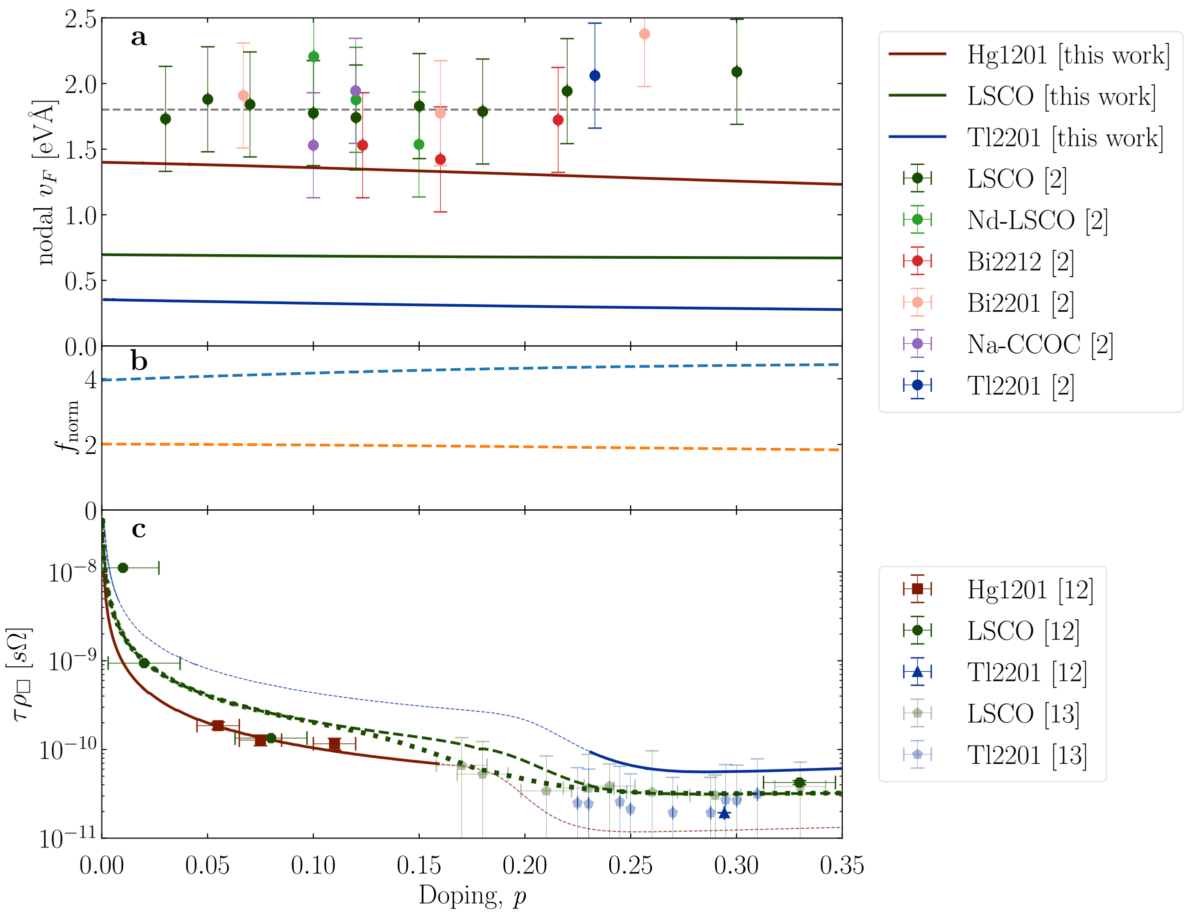


**Figure S3.** **a** The nodal Fermi velocity $v_F$. Full points are experimental data reproduced from Ref. 2 with a mean error of 0.4 eV Å. Clearly $v_F$ is, within error bars, compound- and doping-independent. Lines depict the derivative of the electronic dispersion based on the tight-binding models in Refs. 3, 8 (Hg1201), Refs. 9, 10 (Tl2201) and Ref. 11 (LSCO). Clearly, the tight-binding $v_F$'s strongly deviate from the experimentally established universal value, which greatly affects the absolute value of the calculated resistivity. To control this imprecision of the tight-binding procedure, the normalization factor $f_{\mathrm{norm}}$ is introduced to set the values of Tl2201 and LSCO nodal "tight-binding" $v_F$ to that of Hg1201. **b** Normalizing factors $f_{\mathrm{norm}}$ between the nodal $v_F$'s of Tl2201 and Hg1201 (blue dashed curve) as well as the nodal $v_F$'s between LSCO and Hg1201 (orange dashed curve). **c** The as-calculated $\tau\rho_\square$'s are compared to the experimentally obtained values by combining $\rho_\square$ from Refs. 12, 13, and $\tau$ from Ref. 14. To obtain Fig. 4 of the main text, we have simply multiplied the calculated $\tau\rho_\square$ values by the corresponding normalization factors from panel **b**.

## References


1. Tsukada, I. & Ono, S. Negative Hall coefficients of heavily overdoped $La_{2-x}Sr_xCuO_4$. *Phys. Rev. B* **74**, 134508, DOI: 10.1103/PhysRevB.74.134508 (2006). Publisher: American Physical Society.
2. Zhou, X. J. *et al.* Universal nodal Fermi velocity. *Nature* **423**, 398–398, DOI: 10.1038/423398a (2003).
3. Vishik, I. M. *et al.* Angle-resolved photoemission spectroscopy study of $HgBa_2CuO_{4+\delta}$. *Phys. Rev. B* **89**, 195141, DOI: 10.1103/PhysRevB.89.195141 (2014). Publisher: American Physical Society.
4. Vishik, I. M. *et al.* ARPES studies of cuprate Fermiology: superconductivity, pseudogap and quasiparticle dynamics. *New J. Phys.* **12**, 105008, DOI: 10.1088/1367-2630/12/10/105008 (2010).
5. Ando, Y., Kurita, Y., Komiya, S., Ono, S. & Segawa, K. Evolution of the Hall coefficient and the peculiar electronic structure of the cuprate superconductors. *Phys. Rev. Lett.* **92**, 197001, DOI: 10.1103/PhysRevLett.92.197001 (2004).
6. Padilla, W. J. *et al.* Constant effective mass across the phase diagram of high-$T_c$ cuprates. *Phys. Rev. B* **72**, 060511, DOI: 10.1103/PhysRevB.72.060511 (2005).
7. Yoshida, T. *et al.* Systematic doping evolution of the underlying Fermi surface of $La_{2-x}Sr_xCuO_4$. *Phys. Rev. B* **74**, 224510, DOI: 10.1103/PhysRevB.74.224510 (2006). Publisher: American Physical Society.
8. Das, T. **Q**=0 collective modes originating from the low-lying Hg-O band in superconducting $HgBa_2CuO_{4+\delta}$. *Phys. Rev. B* **86**, 054518, DOI: 10.1103/PhysRevB.86.054518 (2012). Publisher: American Physical Society.
9. Platé, M. *et al.* Fermi surface and quasiparticle excitations of overdoped $Tl_2Ba_2CuO_{6+\delta}$. *Phys. Rev. Lett.* **95**, 077001, DOI: 10.1103/PhysRevLett.95.077001 (2005). Publisher: American Physical Society.
10. Peets, D. C. *et al.* $Tl_2Ba_2CuO_{6+\delta}$ brings spectroscopic probes deep into the overdoped regime of the high-$T_c$ cuprates. *New J. Phys.* **9**, 28–28, DOI: 10.1088/1367-2630/9/2/028 (2007). Publisher: IOP Publishing.
11. Yoshida, T. *et al.* Low-energy electronic structure of the high-$T_c$ cuprates $La_{2-x}Sr_xCuO_4$ by angle-resolved photoemission spectroscopy. *J. Physics: Condens. Matter* **19**, 125209, DOI: 10.1088/0953-8984/19/12/125209 (2007). Publisher: IOP Publishing.
12. Barišić, N. *et al.* Universal sheet resistance and revised phase diagram of the cuprate high-temperature superconductors. *Proc. Natl. Acad. Sci.* **110**, 12235–12240, DOI: 10.1073/pnas.1301989110 (2013). Publisher: National Academy of Sciences Section: Physical Sciences.
13. Hussey, N. E., Gordon-Moys, H., Kokalj, J. & McKenzie, R. H. Generic strange-metal behaviour of overdoped cuprates. *J. Physics: Conf. Ser.* **449**, 012004, DOI: 10.1088/1742-6596/449/1/012004 (2013). Publisher: IOP Publishing.
14. Barišić, N. *et al.* Evidence for a universal fermi-liquid scattering rate throughout the phase diagram of the copper-oxide superconductors. *New J. Phys.* **21**, 113007, DOI: 10.1088/1367-2630/ab4d0f (2019). 1507.07885.
